# Spatiotemporal associations between the built environment and carbon emission reduction potential of shared on-demand transit

**DOI:** 10.3389/fpubh.2026.1927997

**Published:** 2026-10-20

**Authors:** Xiaofan Wei, Zhe Zhang, Chao Li, Meigen Xue, Hongdi He

**Affiliations:** 1 Center for Intelligent Transportation Systems and Unmanned Aerial Systems Applications Research, State Key Laboratory of Ocean Engineering, School of Naval Architecture, Ocean and Civil Engineering, Shanghai Jiao Tong University, Shanghai, China; 2 School of Vehicle and Mobility, Tsinghua University, Beijing, China; 3 School of Transportation Engineering, Chang'an University, Xi'an, China; 4 Shanghai Urban-Rural Construction and Transportation Development Research Institute, Shanghai, China

**Keywords:** built environment, carbon emission reduction, shared on-demand transit, sustainable transportation, urban mobility

## Abstract

Shared on-demand transit has emerged as a promising mobility solution for reducing transport-related carbon emissions and supporting more sustainable urban environments. However, limited evidence exists on how built environment characteristics are associated with its carbon emission reduction potential across space and time. This study aimed to quantify the maximum carbon emission reduction achievable through ODT and identify the built environment factors associated with its spatial variation. An integer linear programming model combined with a multi-passenger travel optimization framework was developed to estimate achievable carbon emission reductions using travel demand data. An XGBoost model was then used to examine their relationships with built environment characteristics, while SHAP and partial dependence analyses were employed to interpret nonlinear, threshold, and spatially heterogeneous relationships. The results showed that ODT could reduce carbon emissions by up to 17.67% in the morning and 14.48% in the evening. Built environment characteristics exhibited pronounced spatiotemporal heterogeneity, with employment- and education-related land uses showing higher nonlinear thresholds in the evening than in the morning. Local interpretation further revealed substantial spatial variation in feature contributions, with areas surrounding educational facilities, employment centers, and suburban metro stations associated with greater carbon emission reduction potential. These findings provide spatial evidence for targeted ODT implementation and highlight the potential of built environment characteristics to inform low-carbon mobility strategies and sustainable urban transport planning.

## 1 Introduction

Urban transportation systems are among the major contributors to global carbon emissions and therefore play a critical role in achieving sustainable urban development goals (1, 2). In the context of increasing pressure to decarbonize urban mobility while maintaining accessibility and efficiency, shared on-demand transit (ODT) has attracted growing attention as a flexible and demand-responsive mobility solution (3). By consolidating multiple travel requests with similar origins, destinations, and temporal characteristics into shared vehicle trips (4), ODT has the potential to improve vehicle utilization and reduce redundant travel, thereby lowering overall energy consumption and carbon emissions. Rather than operating along fixed routes or schedules, ODT dynamically adjusts service provision in response to observed travel demand patterns, making it particularly suitable for heterogeneous and spatially dispersed urban mobility needs (5). ODT is increasingly regarded as an important component of urban mobility systems, particularly as cities shift toward more flexible, demand-responsive, and low-carbon transportation solutions. By integrating shared mobility principles with dynamic routing and demand matching, ODT has the potential to enhance system efficiency and support more sustainable urban transport development (6).

Existing studies on ODT have primarily focused on service operation and system optimization, including vehicle routing, dynamic ride-matching, fleet scheduling, and demand-responsive service design. These studies have demonstrated that ODT can improve vehicle utilization efficiency and enhance service flexibility. In parallel, a growing body of research has examined the environmental implications of shared mobility services, particularly their potential to reduce carbon emissions through ride-sharing, increased vehicle occupancy, and reduced vehicle kilometers traveled. However, most existing work evaluates these benefits at the system level or under aggregated travel conditions, focusing on average performance improvements rather than spatially differentiated outcomes (7–9). As a result, current understanding remains limited regarding how the carbon emission reduction potential of ODT varies across heterogeneous urban environments and how such variations may inform spatial deployment strategies.

The built environment has long been recognized as a key determinant of urban travel demand, travel behavior, and transport mode choice (10–12). Previous studies have shown that built environment characteristics, including land-use composition, employment and residential density, transit accessibility, and urban functional diversity, substantially influence when, where, and how people travel (13). As a demand-responsive transport mode, the operational efficiency and environmental performance of ODT are inherently dependent on these travel characteristics. Variations in travel demand and spatial activity patterns directly affect opportunities for passenger matching, trip sharing, and vehicle utilization (14), which ultimately determine the carbon emission reduction potential of ODT. Consequently, the carbon emission reduction potential of ODT is expected to exhibit considerable spatial heterogeneity across different urban environments rather than being uniformly distributed. While the influence of the built environment on urban travel behavior has been well established, far less attention has been paid to how it shapes the spatial distribution of carbon emission reduction potential for ODT. As a result, identifying priority areas for ODT deployment from a carbon emission reduction perspective remains a significant challenge for urban transport planning and spatial decision-making.

Addressing this challenge requires a better understanding of how built environment characteristics influence the spatiotemporal carbon emission reduction potential of ODT and how these insights can support spatial deployment decisions. Previous studies have increasingly examined the role of built environment characteristics in shaping demand-responsive and shared mobility services. Wang et al. (15) investigated how built environment characteristics affect DRT use and system survival, while Wang et al. (16) examined the nonlinear effects of built environment characteristics on customized bus use. Liu et al. (17) further examined the relationship between the built environment and the number of shared trips under a two-person ridesharing scenario. These studies have mainly focused on travel demand, service use, or the extent of ride sharing, while the carbon emission reduction potential of ODT has received less attention. In particular, limited attention has been paid to quantifying the carbon emission reduction potential of ODT and identifying how built environment characteristics influence this potential. To address this gap, this study combines integer linear programming with SHAP-based machine learning. Integer linear programming is used to estimate the achievable carbon emission reduction potential of ODT, while XGBoost and SHAP are employed to identify the importance and nonlinear effects of built environment characteristics on carbon reduction potential. The analysis quantifies the achievable carbon emission reduction potential of ODT and reveals the built environment characteristics associated with its spatial variation, providing insights for low-carbon ODT planning and deployment.

## 2 Literature review

ODT represents shared mobility in which vehicles service multiple passengers, coordinating several pick-up and drop-off locations. Several studies highlighted its environmental advantages based on simulation models. For example, in Markham, Toronto, ODT was introduced for the first-mile travel. Simulations of morning peak demand travel revealed that ODT reduced travel costs by approximately 7% and greenhouse gas (GHG) emissions by nearly 30% relative to car travel (5). Similarly, ODT was piloted in Ragusa, Italy, a small to medium-sized city. Researchers conducted simulations to compare ODT with conventional taxi services under varying fleet sizes, route selection strategies, and other operational parameters. The results showed that ODT can significantly reduce total vehicle mileage in high-demand contexts. However, the study also found that ODT’s average per capita mileage tends to increase under low-demand conditions, indicating that its efficiency is influenced by demand density (7). An agent-based simulation was used to investigate the effects of replacing private car trips with on-demand mobility services in Amsterdam, the Netherlands (18). The findings demonstrated a 43% reduction in vehicle kilometers traveled (VKT), highlighting the potential of ODT in reducing traffic-related emissions. In addition, optimization approaches such as Markov decision processes have been used to study the operational benefits of ODT under alternative service strategies (19). Incorporating ridesharing into ODT services increased the load factor by 50% compared with non-ridesharing scenarios. Moreover, allowing a detour of just 5% led to a significant reduction in fleet mileage, suggesting that strategic operational adjustments can substantially enhance both efficiency and sustainability.

Meanwhile, some literatures examined the relationship between on-demand transportation demand and the built environment. Travel activities and the built environment exhibit nonlinear relationships (20). Dalian’s demand-responsive transit system adopts a flexible routing strategy that is dynamically planned based on passengers’ origin and destination information submitted via mobile platforms. Based on 2 years of actual operational data, a related study systematically investigated the determinants of the sustainable development of demand-responsive transit from a multidimensional built environment perspective. The findings demonstrate that built environment attributes, including population density, residential spatial distribution, and road network structure, exert significant influences on demand of demand-responsive services (15). The Gradient Boosting Decision Tree approach was employed to explore how built environment factors influences the demand for demand-responsive transit. The results reveal a significant nonlinear association between these factors and indicate that demand-responsive transit can effectively complement conventional public transportation services (16). In addition, sharing rates were also investigated. Based on origin–destination pairs, a maximum matching optimization model was employed to pair ridesharing orders and estimate the number of matched requests in the Shanghai metropolitan area. The analysis further examined the effects of ridesharing demand levels and built environment characteristics, revealing pronounced spatiotemporal variations in matching outcomes (17). Machine learning methods were examined the relationship between on-demand mobility service demand and population and travel characteristics in Belleville, using more than 6 months of operational data. The results indicate that deploying vehicles in commercial and industrial areas can increase service utilization and that land use type is an important factor of travel demand (21). Moreover, the adoption of small electric vehicles within shared mobility systems can substantially reduce carbon emissions while promoting sustainable mobility (22).

Although the environmental benefits of ODT have been widely recognized, considerably less attention has been paid to whether and why its carbon emission reduction potential varies across different urban environments. Consequently, it remains unclear where ODT can deliver greater carbon emission reduction and how built environment characteristics contribute to these spatial differences. Addressing this question is essential for moving beyond evaluating the average environmental performance of ODT toward supporting evidence-based spatial deployment for low-carbon urban mobility. This study therefore investigates how built environment characteristics explain the spatiotemporal variation in ODT carbon emission reduction potential, with the aim of identifying priority areas where ODT can deliver greater environmental benefits.

## 3 Data description

The passenger travel data used in this study were obtained from the Shanghai Institute of Urban and Rural Construction and Transportation Development. The study area covers an approximately 11.9 km × 11.8 km area in Zhangjiang, Shanghai, China. The dataset records the geographic coordinates (longitude and latitude) of passenger origins and destinations, together with their corresponding boarding and alighting times. The data used in this study cover 6 days during a week in mid-September 2021, including four weekdays and two weekend days. The study focuses on the morning period from 7 to 10 and the evening period from 17 to 20 to examine the spatial variation in ODT carbon emission reduction potential across different travel periods. During the three-hour morning period, the average daily number of trips was 1,145 on weekdays and 967 on weekends, corresponding to 6,514 trips across the 6 days. During the three-hour evening period, the corresponding averages were 1,292.75 and 1,168 trips per day, respectively, resulting in 7,507 trips. The actual trip distance averaged 5.30 km, with an interquartile range of 2.82–7.08 km. To capture the spatial variation in travel-related carbon emissions and built environment characteristics, the study area was divided into 289 regular 700 m grids, which served as the spatial units of analysis. These records capture both the spatial distribution of travel demand and its temporal dynamics throughout the day. The study area contains a mixture of residential neighborhoods, commercial centers, industrial parks, and research institutions, resulting in diverse land-use patterns and heterogeneous travel demand that provide an appropriate setting for evaluating the performance of ODT. This diversity creates variation in travel demand and built environment characteristics, making the study area suitable for investigating how urban environments influence the carbon emission reduction potential of ODT.

The passenger trip data exhibit pronounced spatiotemporal variability, reflecting differences in travel intensity across locations and time periods. As illustrated in Figure 1, higher concentrations of trip origins and destinations are observed in the eastern and northwestern parts of the study area, indicating spatial clustering of travel demand. Such heterogeneous demand patterns provide favorable conditions for evaluating the carbon emission reduction potential of ODT under different urban environments and examining how variations in the built environment may influence its environmental performance. The road network was obtained from OpenStreetMap, an openly accessible geographic database that has been widely used in transportation research because of its comprehensive road coverage and high positional accuracy. The road network was preprocessed and represented as a connected graph consisting of road intersections and road segments, enabling network-based route calculations. To ensure spatial consistency between passenger trip records and the road network, the origin and destination of each trip were matched to the nearest network nodes. Consequently, all travel paths were generated along the road network, allowing travel distance estimation to better reflect actual roadway conditions and providing a reliable spatial basis for subsequent carbon emission analysis.

**Figure 1:**
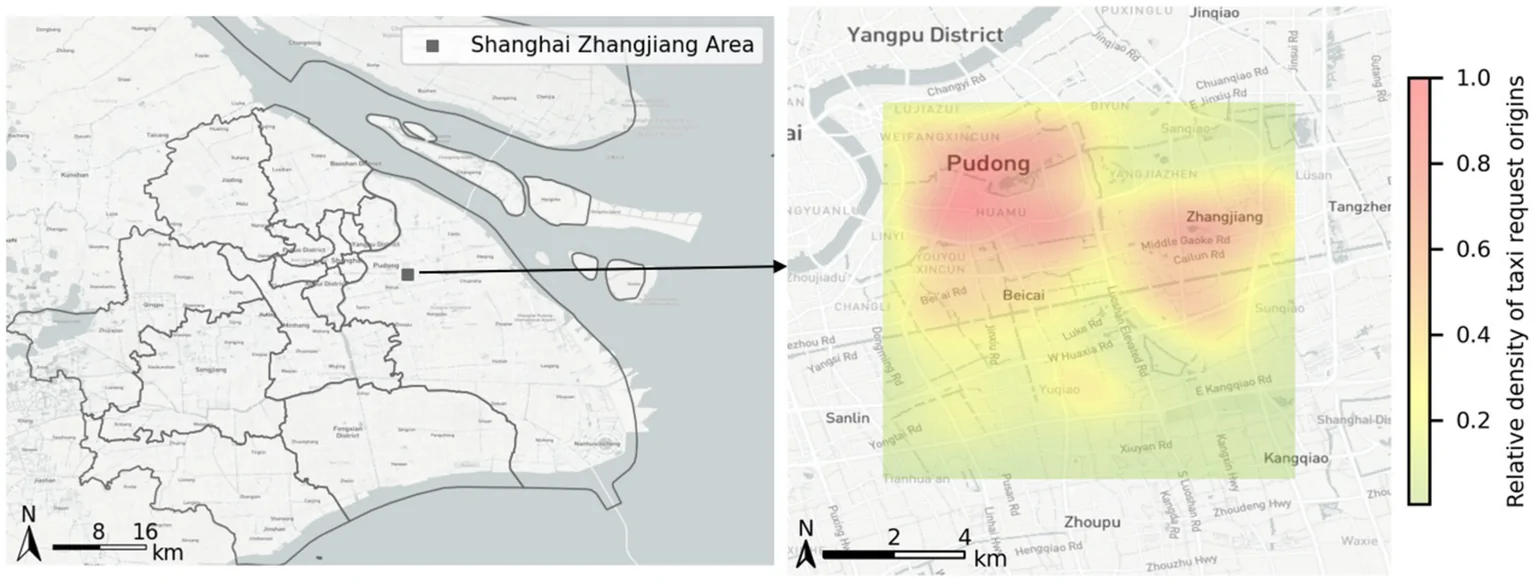
Spatial distribution of trip origins in Shanghai Zhangjiang.

## 4 Methodology

### 4.1 Carbon emission reduction estimation

To estimate the carbon emission reduction potential of ODT, it is first necessary to determine how passenger travel requests can be optimally grouped into shared vehicle trips under realistic operational conditions. Because the environmental performance of ODT depends directly on vehicle routing, passenger matching, and trip sharing efficiency, reliable carbon emission estimation requires an optimized service schedule rather than arbitrary vehicle assignments. Therefore, this study first formulates the ODT dispatching problem as a network-based integer linear programming model to generate feasible shared trips and corresponding vehicle routes prior to carbon emission estimation. The optimization framework operates on a road network in which passenger origins and destinations are first matched to the nearest network nodes. The service system is assumed to operate a fleet of 400 vehicles, each with a total capacity of 8 persons including the driver. Passenger requests are collected every 30 s and processed in batches, with a maximum passenger waiting time of 5 min. Based on the spatial distribution of travel requests and the underlying road network, feasible passenger groups are then constructed by jointly considering vehicle capacity, passenger waiting time, pickup and drop off time windows, travel time, and routing feasibility. The optimization model then determines suitable combinations of vehicle trips, which are used to estimate carbon emissions under the optimized ODT operation.

The optimization objective is to maximize the total service performance of the selected candidate plans, with priority given to serving more passengers while reducing their in-vehicle delay. The objective function is formulated as Equation 1:


$$\text{maxZ}=\sum_{p\in C}(\mathrm{MN}-D)x_{p}$$ (1)

where $x_{p}$ is the binary decision variable for candidate service plan p , with $x_{p}=1$ if plan p is selected and $x_{p}=0$ otherwise. N denotes the number of passengers served by candidate plan p , and D denotes the total in-vehicle delay of the passengers served by that plan. M is a sufficiently large weighting coefficient that prioritizes passenger service over in-vehicle delay. Thus, the objective first prioritizes serving more passengers and then seeks to reduce their in-vehicle delay. To ensure that each vehicle is assigned exactly one candidate service plan at the current dispatching time, the following constraint is imposed by Equation 2:


$$\sum_{p\in C_{v}}x_{p}=1\forall v\in V$$ (2)

where $C_{v}$ denotes the set of candidate service plans generated for vehicle v . For each passenger request, at most one selected candidate service plan can serve the request. This constraint is imposed by Equation 3:


$$\sum_{p\in C_{r}}x_{p}+y_{r}=1\forall r\in R$$ (3)

where $C_{r}$ denotes the set of candidate service plans containing passenger request r , and $y_{r}$ is a binary variable indicating whether passenger r is unserved. Thus, $y_{r}=0$ when passenger r is served by a selected candidate plan and $y_{r}=1$ when no selected candidate plan serves the passenger.

After solving the linear optimization model using Gurobi 11.0, detailed travel metrics are obtained for each passenger, including the shortest possible travel path, the simulated travel path under the shared mobility service, the corresponding carbon emissions along the shortest path, and the actual carbon emissions incurred during the trip. Carbon emissions are calculated by first estimating the energy consumption of each vehicle over its route segments according to the specified energy consumption rate. For electric vehicles, the resulting energy consumption is converted into carbon emissions using an emission factor based on the local electricity generation mix. When multiple passengers share the same vehicle and travel along a common route segment, the emissions associated with that segment are proportionally allocated among all passengers on board. The passenger-level carbon emissions are then obtained by summing the allocated emissions across all route segments traveled by each passenger. Each passenger’s total carbon emissions are calculated by summing their share of emissions across all segments of their actual travel path. The carbon emission of passenger i is calculated as Equation 4:


$$E_{i}=\sum_{s\in S_{i}}(\frac{e_{s}}{n_{s}})$$ (4)

where *S_i_* is the set of all route segments traveled by passenger *i*, *n_s_* is the number of passengers onboard the vehicle during segment *s*, *e_s_* is the total carbon emissions on segment *s*, *e_s_*​ is calculated as Equation 5:


$$e_{s}=d_{s}\cdot e_{c}\cdot \mathrm{EF}$$ (5)

where *d_s_* is the length of segment *s*, *e_c_* refer to energy consumption per kilometer (e.g., kWh/km), *EF* denotes the electricity-related carbon emission factor (e.g., kgCO₂/kWh). Since the carbon emission changes considered in this study are primarily associated with passenger travel, carbon emissions are estimated based on the road segments traveled with passengers onboard. Therefore, empty mileage and vehicle repositioning trips are not included in the emission calculations. As a result, the estimated carbon emission reduction potential reflects only changes in passenger-serving travel and may be somewhat overestimated if additional empty vehicle movements are required in practice. Nevertheless, this assumption enables a consistent comparison of travel-related emissions across scenarios and captures the direct changes associated with passenger travel demand.

The carbon emission reduction can be calculated as Equation 6:


$$\Delta E_{i}=E_{i,T}-E_{i,O}$$ (6)

where *E_i, T_* is carbon emissions of passenger i for taxi, *E_i, O_* is carbon emissions of passenger i for ODT service. Energy consumption (*EC*) is specified as 0.2kWh/km (23, 24). The electricity emission factor (*EF*) is set at 0.548 kgCO₂ per kWh based on the 2020 carbon dioxide emission factor for the Shanghai regional power grid (25). This factor is derived from the composition of multiple electricity sources, such as thermal power and wind power, together with interregional electricity exchanges and electricity consumption data. Although the study data were collected in September 2021, the 2020 emission factor is adopted for carbon emission estimation in this study given its temporal proximity to the study period. The procedure for estimating carbon emission reductions is presented in Figure 2.

**Figure 2:**
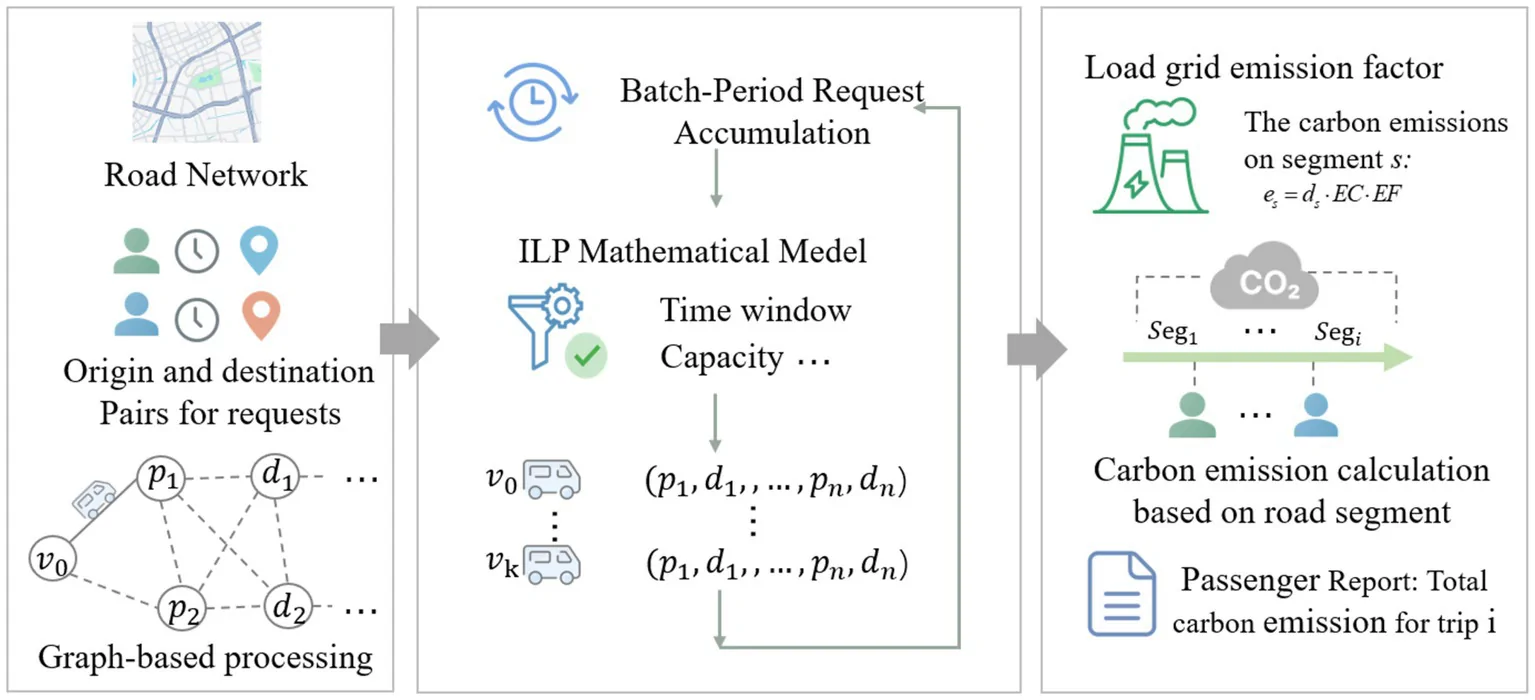
Framework of carbon emission reduction estimation.

### 4.2 Built environment characterization

To capture the spatial variation in travel-related carbon emissions and built environment characteristics, the study area was divided into regular grids. Grid-based spatial analysis has been widely adopted in previous studies of ridesplitting and transportation emissions to examine the spatial distribution of emissions and their influencing factors (26, 27). The selection of this spatial scale was informed by Liu et al. (28), who conducted a multi-scale spatial analysis in central Shanghai and found that spatial differentiation in urban functional mix became weaker as the grid size increased, while a relatively fine spatial scale was more effective in preserving neighborhood-level spatial variation. These findings provide a relevant spatial-scale reference for the present study, given the similar urban context. Accordingly, this spatial scale was adopted to retain local spatial variation while providing a sufficiently broad unit for the consistent aggregation of travel demand, carbon emissions, and built environment characteristics. All trip origins were assigned to their corresponding grid cells, and built environment attributes were calculated at the grid level. Carbon emission reductions were subsequently aggregated to the grid in which each trip originated, enabling a spatially explicit assessment of how built environments indicators affect the carbon emission reduction potential of ODT.

To characterize the spatial heterogeneity of the urban environment, built environment variables were selected according to the widely adopted 5D framework, which describes urban form from the perspectives of Density, Diversity, Design, Destination accessibility, and Distance to transit. This framework has been extensively applied to investigate the relationships between the built environment and travel behavior and provides a systematic basis for examining how urban spatial characteristics influence the carbon emission reduction potential of ODT. All variables were calculated at the grid level and linked to trip origin locations.

The Density dimension represents the concentration of human activities and was measured using population density (residents/km^2^) derived from WorldPop data. Higher population density generally indicates greater travel demand and increases the likelihood of passenger consolidation during peak periods. The Diversity dimension describes the functional composition of urban land use and was characterized by the densities of residential, company, restaurant, education, healthcare, and retail POIs (POIs/km^2^). These variables collectively reflect the spatial distribution of residential, employment, commercial, and public service functions, which influence the generation of travel demand and the opportunities for trip sharing. The Design dimension characterizes the physical structure of the transport network and was represented by road network density, calculated as the total length of road segments per square kilometer using OpenStreetMap data. Greater road network density generally indicates better network connectivity and provides more routing alternatives for shared mobility services. Destination accessibility was represented by the Euclidean distance from each grid centroid to the city center, reflecting the spatial accessibility of major employment and activity centers. The distance to transit dimension was represented by the Euclidean distance from each grid centroid to the nearest metro station. This variable reflects the accessibility of high-capacity public transportation and captures the spatial relationship between ODT services and the existing urban transit network. These variables provide a multidimensional representation of the built environment and enable a systematic investigation of how different urban contexts influence the spatiotemporal carbon emission reduction potential of ODT. This characterization further supports the interpretation of nonlinear relationships, threshold effects, and spatial heterogeneity in the subsequent analysis (Table 1).

**Table 1:** Description of independent variables.

Dimension	Variable	Description	Unit	Data source
Density	Population density	Number of residents per square kilometer	persons/km^2^	WorldPop
Diversity	Land-use mix	Entropy-based land-use mix index calculated from POI categories	-	POI data
	Residential density	Number of residential POIs per square kilometer	POIs/km^2^	POI data
	Company density	Number of company POIs per square kilometer	POIs/km^2^	POI data
	Education density	Number of educational facility POIs per square kilometer	POIs/km^2^	POI data
	Healthcare density	Number of healthcare facility POIs per square kilometer	POIs/km^2^	POI data
	Restaurant density	Number of restaurant POIs per square kilometer	POIs/km^2^	POI data
	Shop density	Number of shop POIs per square kilometer	POIs/km^2^	POI data
Design	Road density	Total road length per square kilometer	km/km^2^	OpenStreetMap
Destination accessibility	Distance to city center	Euclidean distance from the grid centroid to the city center	m	GIS calculation
Distance to transit	Distance to metro station	Euclidean distance from the grid centroid to the nearest metro station	m	Metro station data

### 4.3 Interpretation of built environment effects

This study employs XGBoost model to examine nonlinear influences of built environment characteristics on carbon emission reduction potential of ODT. The method relies on an ensemble of decision trees constructed sequentially to enhance predictive performance. Through iterative error minimization, the model is able to represent complex interactions among explanatory variables.

Formally, given a dataset with n observations and m explanatory variables, the predicted carbon emission reduction potential for observation i is expressed as Equation 7:


$${\hat{y}}_{i}=\sum_{k=1}^{K}f_{k}(x_{i}),f_{k}\in F$$ (7)

where $x_{i}$ denotes the vector of built environment indicators at the origin grid, $f_{k}$ refers to an individual regression tree within the ensemble, *K* specifies the ensemble size, and *F* defines the set of regression trees. Each tree divides the feature space into a collection of non-overlapping regions, with a fixed predictive value associated with each region.

Model interpretability is achieved through SHapley Additive exPlanations (SHAP), which quantify the contribution of individual built environment variables to model predictions. SHAP is grounded in cooperative game theory and attributes each prediction to its constituent features in a consistent manner. Global feature importance is quantified using the mean absolute SHAP value across all samples. Specifically, the global importance of feature j is calculated as Equation 8:


$$I_{j}=\frac{1}{n}\sum_{i=1}^{n}|\phi_{\mathrm{ij}}|$$ (8)

where $I_{j}$ denotes the global SHAP importance of feature j , n is the number of samples, and $\phi_{\mathrm{ij}}$ represents the SHAP value of feature j for sample i . The absolute value is used to measure the magnitude of the contribution of each feature regardless of whether its effect on the prediction is positive or negative. To express the relative importance of each feature as a percentage, the global importance values are normalized using Equation 9:


$$P_{j}=\frac{I_{j}}{\sum_{k=1}^{p}I_{k}}\times 100\%$$ (9)

where $P_{j}$ denotes the percentage global importance of feature j , and p is the total number of features.

It captures the aggregate effect of each built environment indicator, and supports an assessment of carbon emission reduction potential between morning and evening travel periods. While SHAP explains feature contributions at the individual observation level, Partial Dependence Plots (PDPs) are employed to illustrate the average effect of a single feature on model predictions, with the effects of other variables held constant. For a feature, the partial dependence function is defined as Equation 10:


$$\mathrm{PDP}(x_{s})=\frac{1}{n}\sum_{i=1}^{n}f(x_{s},x_{i,-s})$$ (10)

where $x_{i,-s}$ denotes all features except $x_{s}$ , PDPs uncover nonlinear trends and threshold effects in how built environment affect carbon emission reduction potential. In addition, two-dimensional PDPs are employed to investigate interaction effects between pairs of features, enabling the identification of synergistic relationships.

## 5 Results and discussion

### 5.1 Spatiotemporal distribution of carbon emission reduction potential

Figure 3 presents the spatiotemporal distribution of carbon emission reduction aggregated at the grid level, with the estimated reduction associated with each trip assigned to its origin grid. The results show a 17.67% reduction in carbon emissions during the morning travel period and a 14.48% reduction during the evening travel period under ODT service. At the trip level, the average emission reduction is 100.54 g per trip in the morning travel period and 82.83 g per trip in the evening travel period, indicating considerable carbon emission reduction associated with shared ODT services during these travel periods. Beyond the overall reduction magnitude, Figure 3 reveals pronounced spatial heterogeneity in carbon emission reduction potential across the study area. During the morning peak, higher carbon emission reduction potential is primarily concentrated in the southwestern and southeastern areas, whereas lower or even negative values are observed in several northern grids. In contrast, during the evening peak, the spatial pattern shifts toward the northwestern part of the study area, indicating that areas with greater carbon emission reduction potential differ substantially between time periods. These findings indicate that priority deployment of ODT should consider local spatiotemporal characteristics rather than relying on a uniform implementation strategy across the study area.

**Figure 3:**
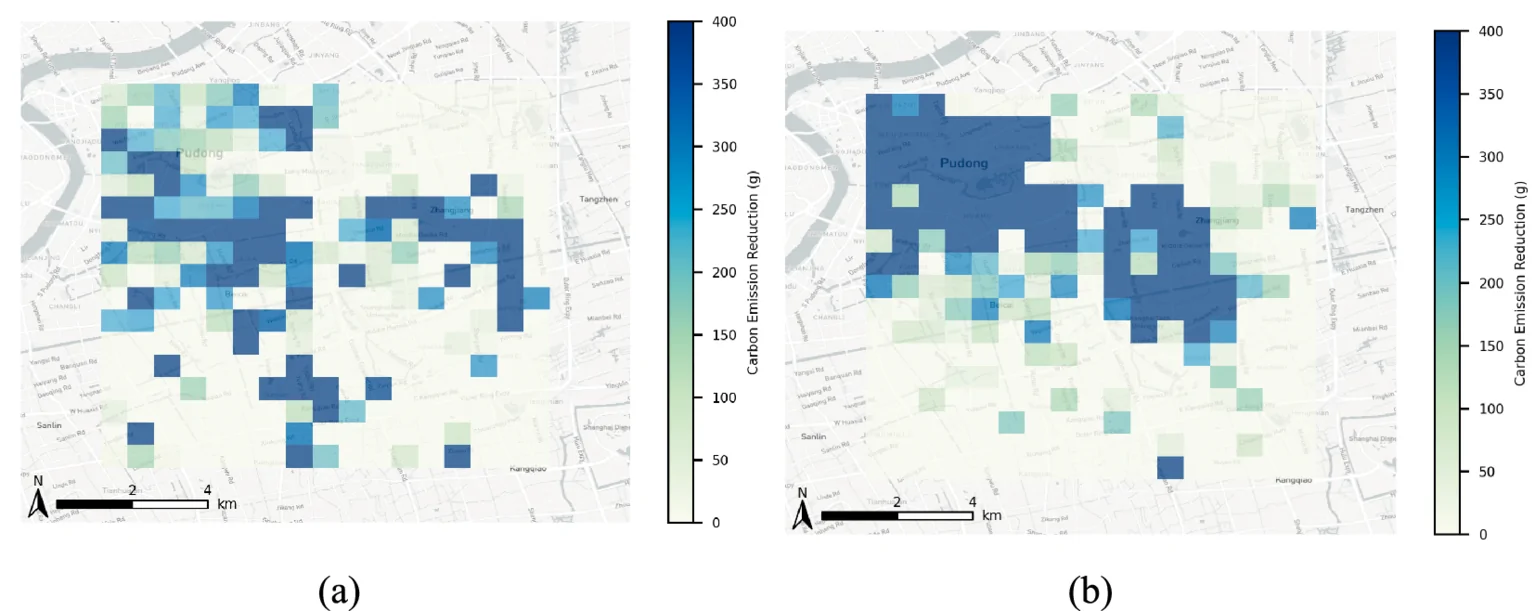
Spatiotemporal distribution of carbon emission reduction. **(a)** Morning travel period; **(b)** Evening travel period.

### 5.2 Relative importance of built environment variables

To quantify the relative contribution of built environment characteristics to the spatial variation in ODT carbon emission reduction potential, the trained XGBoost model was interpreted using feature importance analysis. The XGBoost model was configured with 120 estimators, a learning rate of 0.02, and a maximum tree depth of 4. The model was trained using 80% of the observations, while the remaining 20% were retained as an independent test set. On the training set, the model achieved an R^2^ of 0.88, an RMSE of 1459.03, and an MAE of 1033.62. On the independent test set, the model achieved an R^2^ of 0.64, with an RMSE of 2116.47 and an MAE of 1419.95. The lower performance observed on the test set indicates some loss of generalization relative to the training set; nevertheless, the model retained a reasonable predictive capability on unseen observations, suggesting that it captures substantial variation in carbon emission reduction potential. To further assess whether spatial dependence among neighboring grids could affect the reliability of the model results, Global Moran’s I was calculated for the model residuals. Spatial dependence may lead to correlated residuals and potentially affect the reliability of model interpretation. A Queen contiguity spatial weight matrix was therefore used to evaluate the spatial autocorrelation of the residuals. The residuals yielded a Moran’s I of 0.0521 (*p* = 0.255). Although the Moran’s I value was positive, it was not statistically significant at the 5% level, indicating that no statistically significant spatial autocorrelation was detected in the model residuals. While this result supports the reliability of model interpretation, potential spatial leakage associated with geographically adjacent observations under the random sampling strategy cannot be completely ruled out. Given the overall predictive performance and the limited residual spatial dependence observed, the trained model was subsequently used to examine the relative importance of built environment variables. Figure 4 presents the global importance of each built environment variable during the morning and evening travel periods.

**Figure 4:**
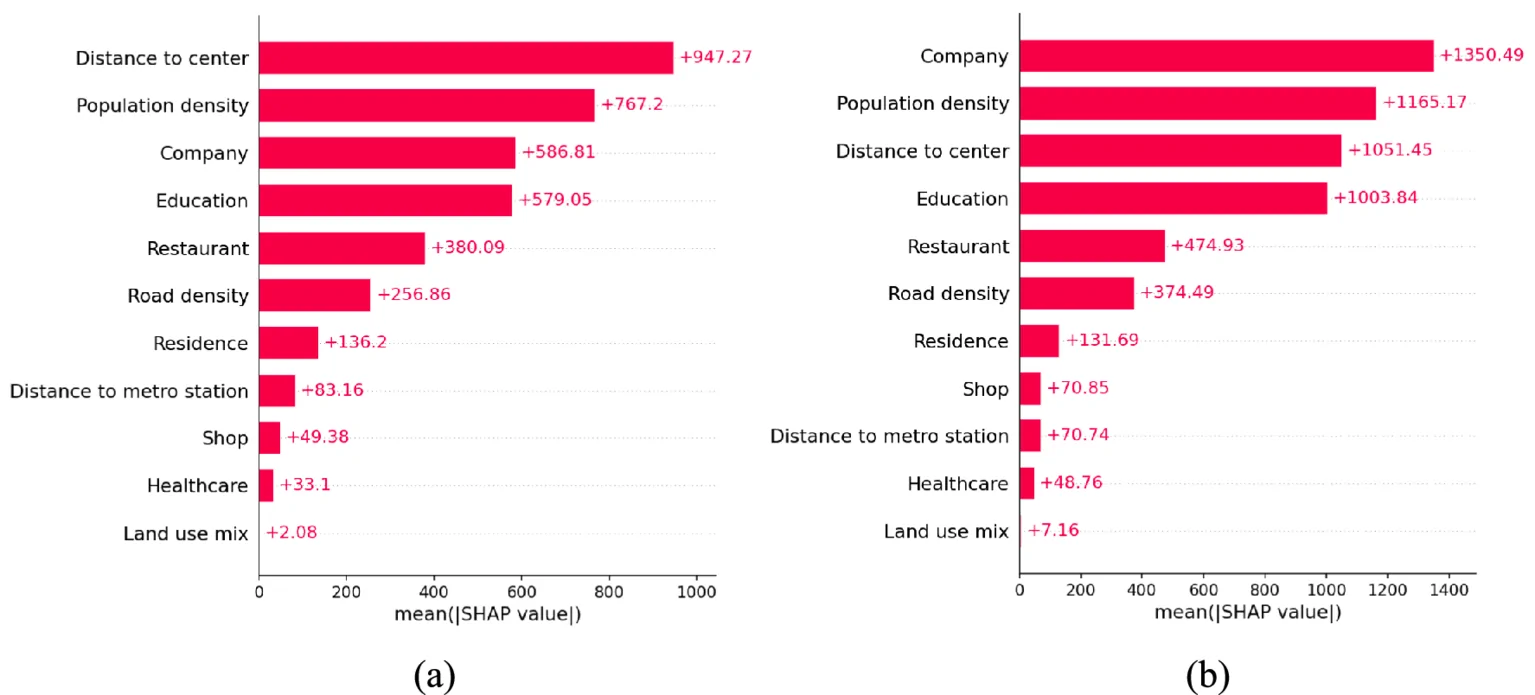
Built environment factor importance across morning and evening travel periods. **(a)** Morning travel period; **(b)** Evening travel period.

During the morning travel period, distance to the urban center showed the highest normalized SHAP importance (24.79%), followed by population density (20.08%), company density (15.36%), and education density (15.15%). Restaurant density (9.95%) and road density (6.72%) also showed moderate importance, whereas residence density (3.56%) and other variables exhibited smaller importance values. These results suggest that morning carbon emission reduction potential is associated with urban spatial structure and the concentration of population and activities, reflecting the directional characteristics of commuting demand. During the evening travel period, company density exhibited the highest normalized SHAP importance (23.49%), followed by population density (20.27%), distance to the urban center (18.29%), and education density (17.46%). Restaurant density (8.26%) and road density (6.51%) also showed moderate importance, whereas the remaining variables exhibited relatively smaller importance values. Compared with the morning period, the higher importance of company density indicates that evening carbon emission reduction potential is more closely associated with the spatial distribution of workplaces and surrounding activities.

The differences in feature importance between the morning and evening periods suggest that the associations between the built environment and ODT carbon emission reduction potential exhibit temporal heterogeneity. Variables representing the overall urban spatial structure, particularly distance to the urban center and population density, remain consistently important across both periods, highlighting their fundamental role in shaping travel demand and opportunities for ride sharing. In contrast, the importance of specific land-use functions changes with travel purposes and activity patterns over the course of the day. These findings suggest that effective deployment of ODT should account for both temporal variations in travel demand and local built environment characteristics, rather than adopting a uniform deployment strategy across the entire urban area.

### 5.3 Nonlinear effects of built environment variables

To further examine how individual built environment characteristics are associated with carbon emission reduction potential, partial dependence plots (PDPs) were employed to characterize their nonlinear relationships with the predicted outcomes. Figures 5, 6 illustrate the partial dependence of the predicted carbon emission reduction potential on individual variables while averaging over the distribution of the other variables. The results reveal pronounced nonlinear patterns, suggesting that the relationships between built environment characteristics and carbon emission reduction potential cannot be adequately represented by simple linear associations. Instead, several variables exhibit clear threshold patterns, beyond which their associations with carbon emission reduction potential change substantially. Moreover, the nonlinear relationships differ between the morning and evening periods, suggesting temporal heterogeneity in the associations between the built environment and carbon emission reduction potential.

**Figure 5:**
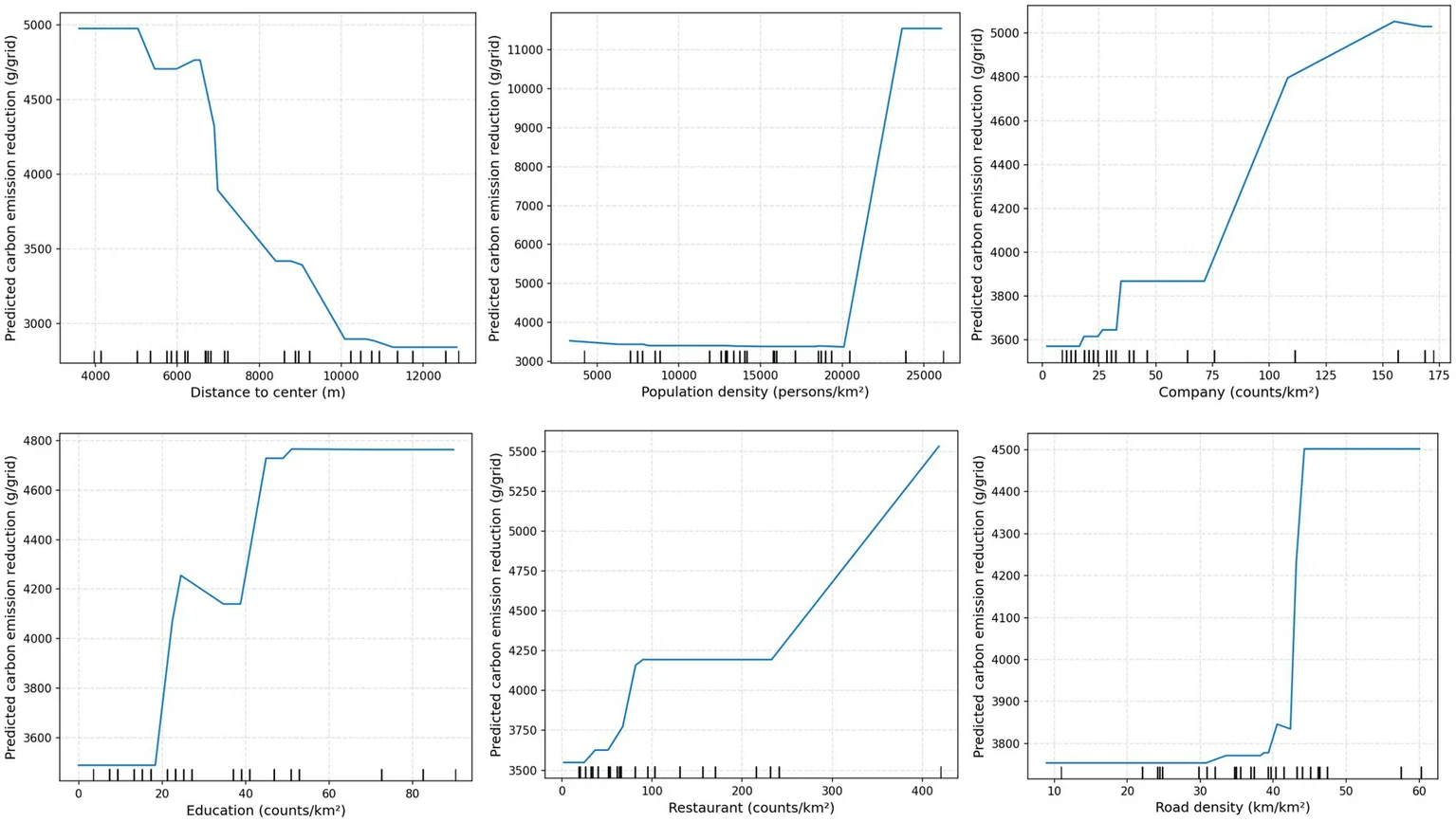
Partial dependence plot in the morning travel period.

**Figure 6:**
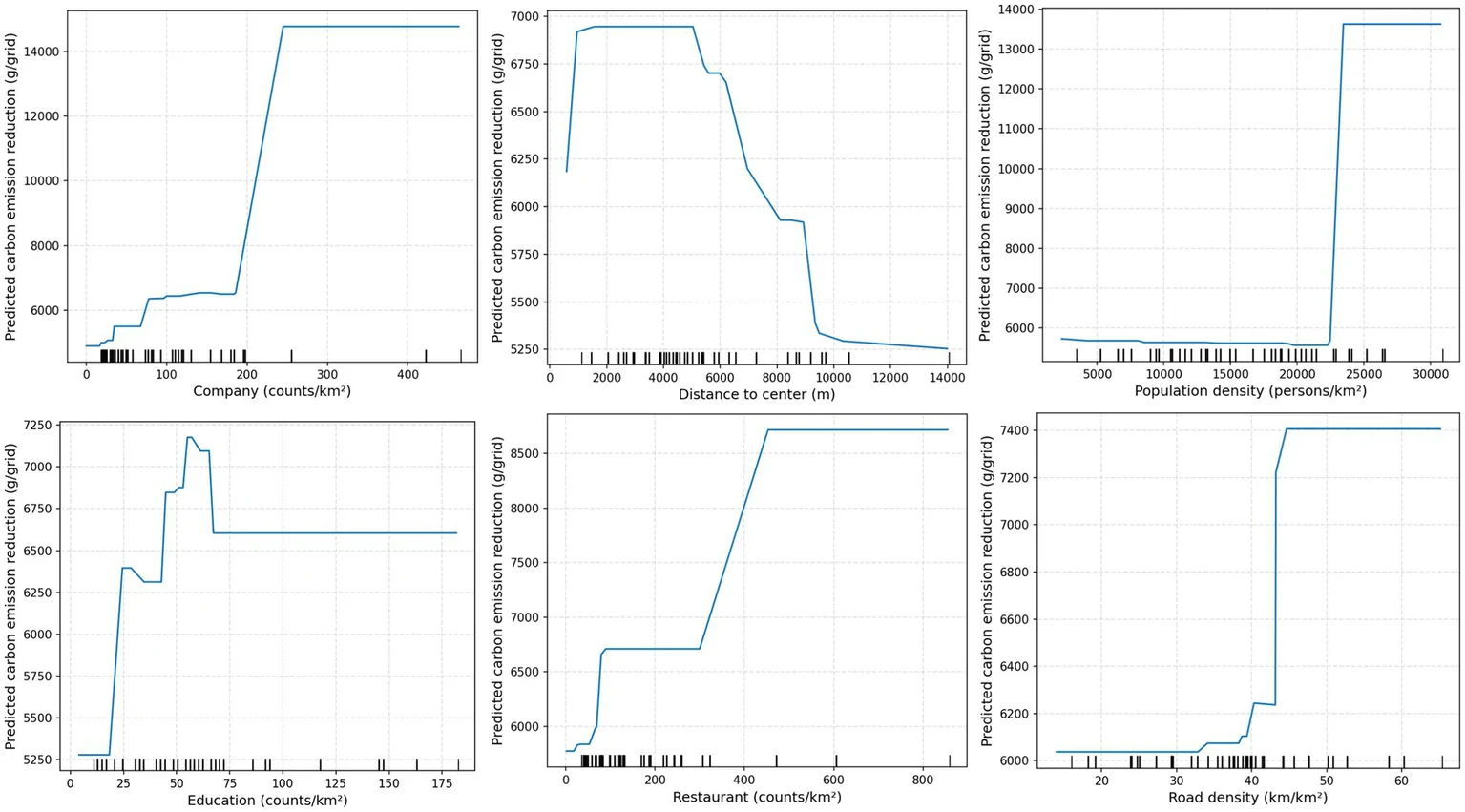
Partial dependence plot in the evening travel period.

During the morning travel period, several built environment variables exhibit distinct nonlinear relationships with carbon emission reduction potential. Distance to the urban center shows a pronounced negative relationship, with carbon emission reduction potential declining substantially beyond approximately 7 km from the city center. Population density demonstrates an increasing pattern, with carbon emission reduction potential rising rapidly at densities of around 20,000 persons/km^2^ before gradually approaching a plateau. Company density also exhibits an increasing trend, with the modeled carbon emission reduction potential appearing to rise more noticeably at values above approximately 75 companies/km^2^. Education facility density shows a similar pattern, with the PDP indicating a more pronounced increase at densities above approximately 40 facilities/km^2^. Restaurant density likewise exhibits an upward pattern at relatively high values, with carbon emission reduction potential increasing around 200 restaurants/km^2^. Road network density exhibits an increasing nonlinear relationship, with a more rapid increase in carbon emission reduction potential observed near 40 km/km^2^. Overall, these results suggest that ODT may have greater carbon emission reduction potential in dense urban environments with diverse activities and good connectivity.

During the evening travel period, the nonlinear relationships remain evident but exhibit several notable differences. Company density continues to be an important variable, with the PDP suggesting that carbon emission reduction potential appears to increase more noticeably at values above approximately 180 companies/km^2^. Population density also exhibits a positive nonlinear relationship, with carbon emission reduction potential rising more rapidly at densities above approximately 22,000 persons/km^2^. In contrast to the morning period, carbon emission reduction potential remains relatively stable at distances of approximately 6 km or less from the city center before declining more rapidly at greater distances. Education facility density is associated with higher carbon emission reduction potential over a range of approximately 40–70 facilities/km^2^, followed by a slight decline at higher densities. Restaurant density likewise shows a more noticeable increase in carbon emission reduction potential at relatively high values, with the PDP suggesting an increase above approximately 300 restaurants/km^2^. Road network density shows a similar nonlinear relationship to that observed during the morning period. Compared with the morning period, several built environment variables exhibit changes in the locations and shapes of their nonlinear relationships in the evening, suggesting that the associations between these variables and carbon emission reduction potential vary with the spatial distribution of travel demand.

The identified nonlinear relationships suggest that the associations between built environment characteristics and ODT carbon emission reduction potential cannot be adequately captured by simple linear assumptions. Instead, most variables exhibit distinct threshold patterns, with their associations with carbon emission reduction potential changing noticeably across different levels of the built environment. In particular, the relationships observed for urban density, land-use intensity, and transport connectivity indicate that their associations with carbon emission reduction potential vary before and after specific threshold levels, with the strength of these associations generally becoming weaker beyond the identified thresholds. The temporal shifts in threshold values further suggest that the relationships between built environment characteristics and carbon emission reduction potential vary across travel periods with different demand characteristics, resulting in distinct patterns between the morning and evening periods. Overall, these findings provide a more nuanced understanding of the nonlinear and temporally heterogeneous associations between the built environment and ODT carbon emission reduction potential.

From a planning perspective, these findings suggest that ODT deployment should be tailored to local built environment conditions rather than implemented uniformly across the city. Areas characterized by relatively high population density, concentrated employment, diverse urban functions, and well-connected road networks generally provide more favorable conditions for achieving greater carbon emission reduction potential. Moreover, the observed temporal differences indicate that deployment strategies should account for variations in travel demand between the morning and evening periods. Integrating these nonlinear relationships into transport planning can help prioritize areas where ODT is most likely to generate substantial environmental benefits while supporting low-carbon urban mobility and sustainable urban development.

### 5.4 Interaction effects of built environment characteristics on carbon emission reduction potential

While the global importance and partial dependence analyses characterize the associations between individual built environment variables and carbon emission reduction potential, these relationships may also vary with other built environment characteristics. To examine such interaction patterns, two-dimensional partial dependence plots were used to assess how the predicted carbon emission reduction potential varies across combinations of two built environment variables. The interaction pairs were selected based on two considerations. First, distance to the urban center was identified as one of the most important variables in both the morning and evening travel periods according to the global importance analysis. Second, company density and education density represent two major generators of commuting travel demand within the study area and showed pronounced nonlinear relationships with carbon emission reduction potential in the previous analysis. Their interactions with distance to the urban center were therefore examined to assess whether the relationship between distance to the urban center and carbon emission reduction potential varies across different levels of employment and educational activity density, particularly between central and peripheral locations.

The interaction between distance to center and company density reveals a heterogeneous relationship with carbon emission reduction. Carbon emission reduction increases with company density in both central and peripheral areas, but the increase is more pronounced in areas farther from the urban center. When company density increases from low levels to approximately 200, carbon emission reduction rises from around 4,739 g to 12,094 g when distance to center is less than 7,000 m, compared with an increase from around 2,900 g to 12,094 g when distance to center exceeds 7,000 m. This pattern suggests that peripheral areas exhibit a stronger response of carbon emission reduction to employment concentration. From a planning perspective, areas with relatively high employment concentration in peripheral locations may therefore represent priority areas for ODT deployment. A similar spatially heterogeneous pattern is observed in the interaction between distance to center and education density. When the distance to the center is less than 7,000 m, increasing education density from 0 to approximately 80 is associated with an increase in the predicted carbon reduction potential from 4,003 g to 4532.63 g. In areas farther than 7,000 m from the urban center, the predicted value increases from 1884.96 g to 3473.50 g over the same range of education density. The larger increase in peripheral areas suggests that the predicted carbon reduction potential is more sensitive to education density in locations farther from the urban center. This finding indicates that educational clusters in peripheral areas may be considered as priority locations when identifying ODT deployment areas.

The observed interaction patterns indicate that the association between activity density and predicted carbon reduction potential varies with urban centrality. In particular, areas farther from the urban center with relatively high concentrations of companies or educational facilities exhibit considerable predicted carbon reduction potential. This finding highlights employment and educational clusters in peripheral areas as potential priority locations for ODT deployment. Considering these interaction patterns in spatial planning may help identify areas where multiple favorable built environment characteristics coexist and inform more targeted ODT deployment for carbon emission reduction (Figure 7).

**Figure 7:**
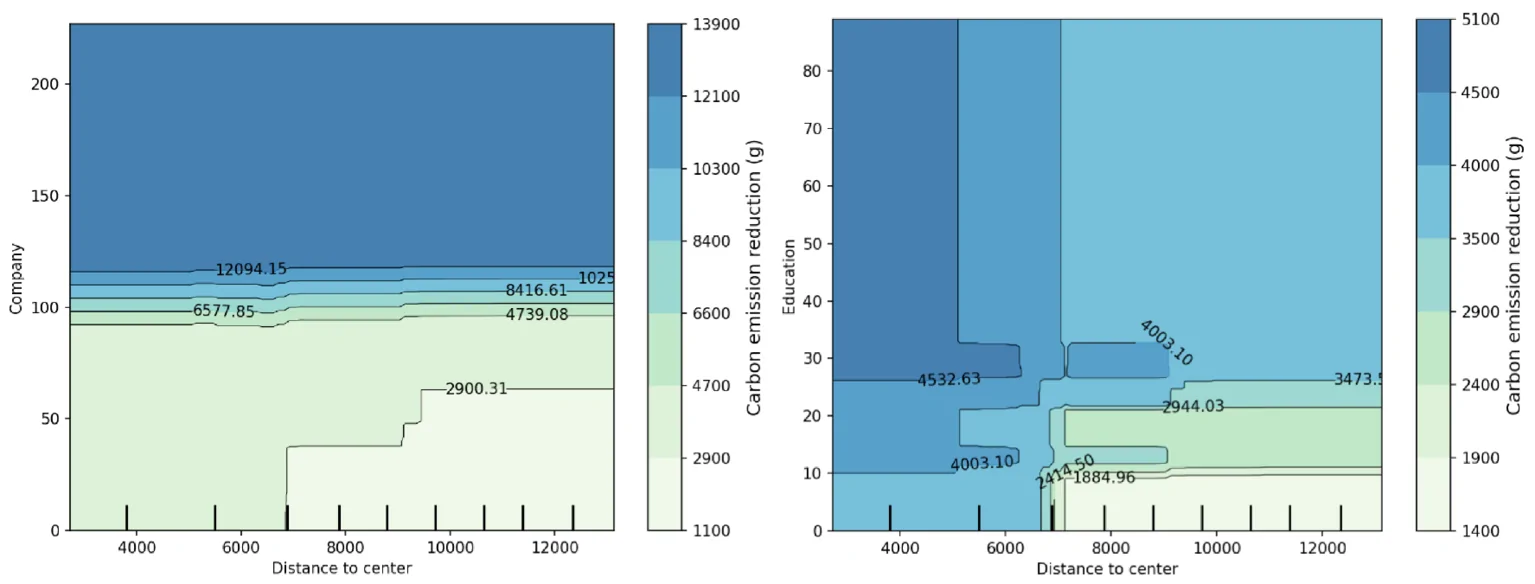
Synergistic impacts among built environment characteristics.

### 5.5 Spatial interpretation of built environment effects in representative urban areas

To further interpret the spatial heterogeneity of built environment effects, this section examines the local contributions of built environment variables using SHAP values. Four representative areas with distinct spatial and built environment characteristics are selected (Figure 8), including a central mixed-use district, a residential-education district, an employment-oriented district, and an outer urban mixed-use district. The four areas were selected based on a comprehensive consideration of their spatial locations and land-use characteristics, representing different urban contexts within the study area. Comparing these areas helps illustrate how the relationships between built environment variables and carbon emission reduction vary across different spatial contexts and provides geographically targeted evidence for ODT deployment.

**Figure 8:**
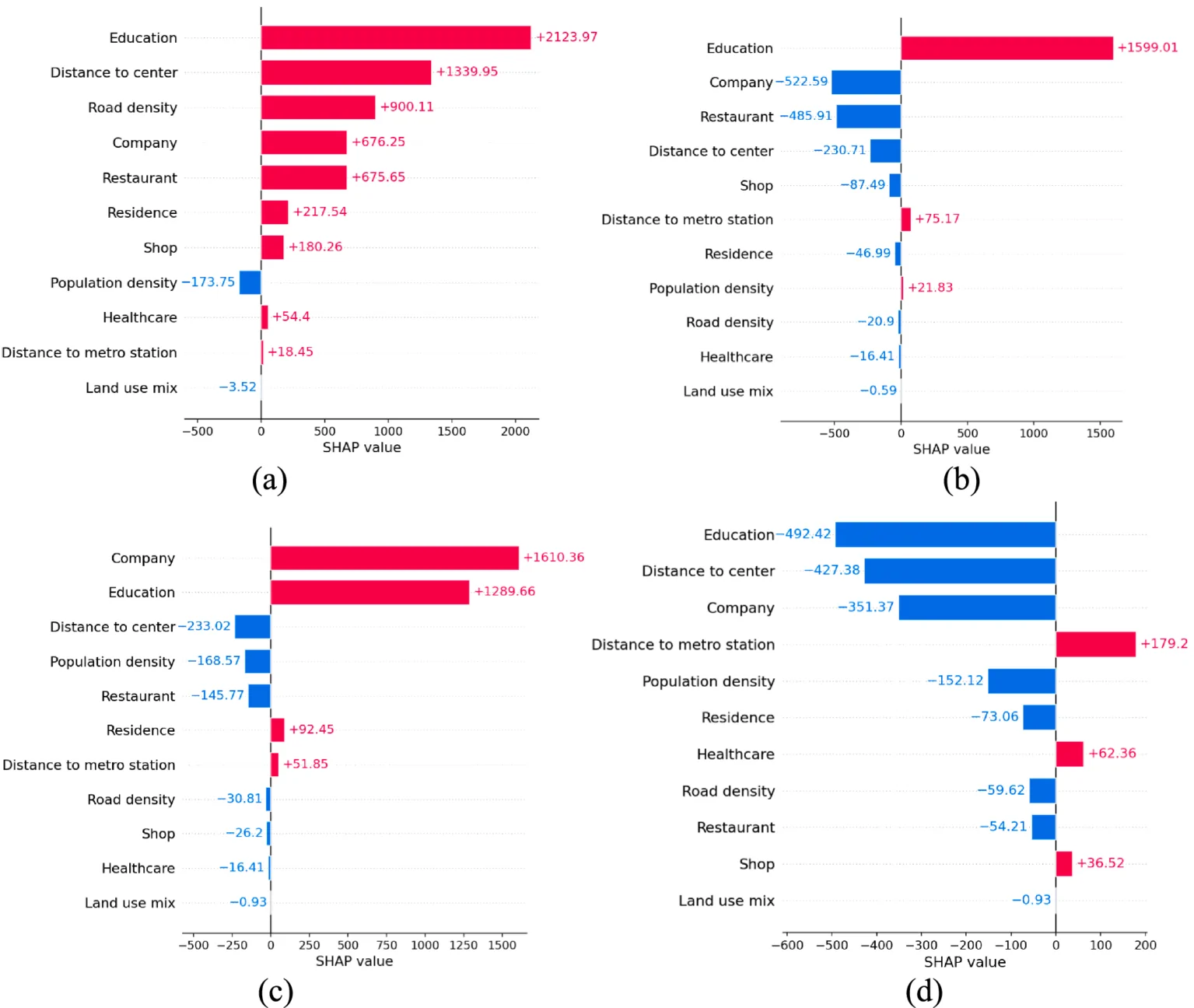
Local SHAP explanations of built environment effects in representative urban areas. **(a)** Central mixed-use area; **(b)** Residential and education area; **(c)** Employment-oriented area; **(d)** Outer urban mixed-use area.

The central mixed-use area represents a compact urban environment characterized by high population density, intensive land-use mixing, and diverse urban functions, including employment, education, commercial activities, residential communities, and healthcare facilities. Local SHAP results show that multiple activity-related variables are associated with carbon emission reduction potential, reflecting the coexistence of diverse travel purposes and concentrated travel demand. Dense road networks and high accessibility are also associated with higher carbon emission reduction potential, indicating favorable conditions for ODT operations in this area. These patterns suggest that ODT may complement existing public transport services in compact urban centers, particularly for short-distance trips, while reducing reliance on individual taxi services. The residential and educational area is located beyond the urban center and is characterized by the coexistence of residential communities and university campuses. Local SHAP results show that education-related and residential variables are strongly associated with carbon emission reduction potential, corresponding to the concentrated travel demand generated by students and nearby residents. Compared with the city center, public transport accessibility is relatively limited, which may increase the relevance of flexible shared mobility services in this area. These characteristics suggest that university-oriented residential districts may represent suitable locations for ODT deployment, where ODT can complement conventional public transport and potentially improve accessibility while reducing carbon emissions.

The employment-oriented area is dominated by office buildings, industrial parks, and innovation facilities, generating concentrated commuting demand during peak periods. Local SHAP results show that employment-related variables dominate carbon emission reduction potential, highlighting the importance of synchronized commuter flows for efficient passenger aggregation. Although metro accessibility is relatively limited, concentrated employment activities provide favorable conditions for ODT services. Therefore, employment clusters located outside high-capacity transit corridors may represent priority areas where ODT can effectively reduce carbon emissions by serving commuting demand. The outer urban mixed-use area is located in the suburban part of the study area and combines mixed land uses with proximity to Metro Line 11. Local SHAP results indicate that metro accessibility, land-use diversity, and commercial activities jointly contribute to carbon emission reduction potential. Rather than supporting concentrated commuting demand, ODT in this context primarily complements public transport by improving first- and last-mile accessibility and facilitating daily activity travel. These findings suggest that suburban mixed-use areas with convenient metro access provide favorable conditions for integrating ODT with existing public transport systems.

Overall, the representative area analysis demonstrates that suitable deployment locations for ODT vary according to local built environment characteristics rather than following a single urban development pattern. High carbon emission reduction potential can be achieved not only in compact urban centers but also in suburban areas with concentrated employment, educational activities, or strong integration with the metro system. These findings emphasize that effective ODT deployment should be tailored to local urban contexts by considering the combined characteristics of land use, accessibility, and travel demand. From a planning perspective, identifying such representative urban environments provides practical guidance for prioritizing ODT deployment and maximizing its contribution to low-carbon urban mobility.

## 6 Conclusion

This study investigates the relationship between built environment characteristics and the spatiotemporal carbon emission reduction potential of shared on-demand transit (ODT), and finds that environmental benefits vary considerably across different urban environments and travel periods. By identifying built environment conditions associated with greater carbon emission reduction potential, the study advances the understanding of how urban form and land-use characteristics are related to the environmental performance of shared mobility services. These findings highlight the importance of considering spatial and temporal context when developing ODT deployment strategies and incorporating built environment characteristics into low-carbon transport planning.

The findings provide several important insights into the spatiotemporal carbon emission reduction potential of ODT. First, ODT shows considerable carbon emission reduction potential during both morning and evening peak periods, although the magnitude and spatial distribution differ between the two periods in association with differences in travel demand patterns. Second, built environment characteristics show strong but temporally heterogeneous associations with carbon emission reduction potential. Population density, employment concentration, and distance to the urban center consistently account for a large proportion of the spatial variation, while their relative importance differs between morning and evening travel periods. Third, the associations between built environment characteristics and carbon emission reduction potential are highly nonlinear and interactive. Threshold and interaction analyses reveal distinct response patterns across different levels and combinations of built environment characteristics, with greater carbon emission reduction potential observed under certain combinations of supportive conditions.

From a planning perspective, the results suggest that ODT deployment should be guided by local built environment characteristics. Areas characterized by concentrated employment activities, and well-developed urban functions provide favorable conditions for efficient passenger sharing and greater carbon emission reduction. In addition, the identified nonlinear thresholds and interaction effects indicate that land-use composition and accessibility should be jointly considered when prioritizing ODT deployment. These findings provide practical evidence for integrating shared mobility services with land-use planning and public transport systems to support low-carbon urban development. Despite these contributions, several limitations should be acknowledged. The temporal coverage of the travel data is relatively short. The OD structure and spatial distribution of travel demand may differ across seasons. These differences could affect trip distances and ODT service efficiency, potentially resulting in variations in the estimated carbon emission reduction potential. Future studies could use longer-term data covering different seasons to better assess these temporal variations. The study focuses on a specific urban area in Shanghai, and the findings may vary across cities with different urban forms, travel demand characteristics, and transportation conditions. Future research could validate the findings across multiple urban contexts to improve their generalizability. In addition, the estimated carbon emissions are subject to uncertainty in the emission factors used in the calculations. Future studies could incorporate more context-specific emission factors to improve the accuracy of emission estimates. Finally, future research could combine machine learning interpretability with causal inference and other empirical evidence to provide stronger support for policy implications.

## Data Availability

The raw data supporting the conclusions of this article will be made available by the authors, without undue reservation.
